# From immune dysregulation to organ dysfunction: understanding the enigma of Sepsis

**DOI:** 10.3389/fmicb.2024.1415274

**Published:** 2024-08-26

**Authors:** Zhi Liu, Yuan Ting, Miao Li, Yukun Li, Yingzheng Tan, Yunzhu Long

**Affiliations:** ^1^Department of Infectious Disease, Graduate Collaborative Training Base of Zhuzhou, Hengyang Medical School, University of South China, Hengyang, China; ^2^Department of Infectious Disease, Zhuzhou Central Hospital, Xiangya Hospital Zhuzhou Central South University, Central South University, Zhuzhou, China; ^3^Jishou University Zhuzhou Clinical College, Medical College, Jishou University, Zhuzhou, China; ^4^Medical College, Jishou University, Xiangxi Tujia and Miao Autonomous Prefecture, Zhuzhou, China; ^5^Department of Assisted Reproductive Centre, Zhuzhou Central Hospital, Xiangya Hospital Zhuzhou Central South University, Central South University, Zhuzhou, China

**Keywords:** Sepsis, neutrophils, T cells, cytokines, molecular mechanism

## Abstract

Sepsis is a syndrome precipitated by immune dysregulation in response to infection, and represents a pivotal factor in global mortality attributed to diseases. The recent consensus delineates sepsis as a perilous state of organ dysfunction arising from the host’s maladaptive reaction to infection. It masks the complexity and breadth of the immune mechanisms involved in sepsis, which is characterized by simultaneous hyperinflammation and immunosuppression. Sepsis is highly correlated with the dysregulation of immune response, which is mainly mediated by various immune cells and their interactions. This syndrome can lead to a plethora of complications, encompassing systemic inflammatory response, metabolic disturbances, infectious shock, MODS, and DIC. Furthermore, more research studies have been conducted on sepsis in the past few years. The pathological characteristics of sepsis have been improved or treated by targeting signaling pathways like NF-B, JAK–STAT, PI3K-Akt, and p38-MAPK. Combined drug therapy is better than single drug therapy for sepsis. This article will review the latest progress in the pathogenesis and treatment of sepsis.

## 1 Introduction

Sepsis denotes a critical impairment of organ function caused by the dysregulated response to infection, posing a substantial challenge not only to clinicians but also to scientific researchers (Gyawali et al., 2019). Inflammatory response, tissue injury, important organ failure, and pathological thrombosis are the main and typical pathophysiological changes in sepsis, indicating infection in the body. Numerous cytokines are released, including TNF-α, interleukins, prostaglandins, and so on (Jacobi, 2002). At the same time, laboratory tests show an increase in white blood cell count, CRP and PCT, elevated lactate levels, and abnormal coagulation function (Fulton et al., 2024). Moreover, the pathogenesis of sepsis remains inadequately understood, potentially attributed to an exaggerated inflammatory reaction and immune suppression stemming from the dysregulation of the reaction to infection (Nedeva et al., 2019). The most common symptoms are fever, elevated heart rate, and hypotension, which may progress to shock, multiple organ failure, and DIC, which are life-threatening (Cawcutt and Peters, 2014).

It is worth noting that prompt recognition and treatment of sepsis are crucial for patient prognosis. Even without a clear diagnosis, initiating anti infection therapy may reduce the incidence rate of sepsis (Sivapalan and Stæhr Jensen, 2015) Because of the different causes and symptoms of sepsis, each patient with sepsis should undergo personalized treatment according to their needs, clinical characteristics, and other parameters in the treatment plan. The aim of treatment is to enhance the patient’s quality of life and avert potential complications (Vincent et al., 2022). Many treatment strategies, including antibiotics, vasoactive drugs, glucocorticoids, and immunomodulatory drugs, are first-line treatments for sepsis, but often need to be paired with other treatment strategies to further improve the clinical symptoms of sepsis (Schmoch et al., 2024).

We summarize the potential molecular mechanisms underlying the pathophysiology of sepsis and the interactions between different cells. In addition, we also summarize the latest developments in potential biomarkers and therapeutic drugs for sepsis, with a view to providing clinicians and researchers with better ideas and current research progress.

## 2 Pathogenesis of sepsis

Sepsis is a syndrome of systemic inflammatory response triggered by infection, with nearly all types of infections capable of causing sepsis. Bloodstream sepsis is a severe infection where pathogens induce systemic inflammatory response through the circulation of blood (Kargaltseva et al., 2022). Moreover, sepsis can also stem from local infectious foci, such as pulmonary infections, abdominal infections, urinary system infections, surgical complications, and other sites that are in communication with the external environment or harbor bacteria themselves (Xie et al., 2020). Bloodstream sepsis often presents with severe inflammatory symptoms such as high fever and chills, progresses rapidly, and carries a high mortality rate (López-Cortés et al., 2017). On the other hand, sepsis arising from local infections may exhibit different symptoms and prognosis depending on the infected site (Mendoza et al., 2022).

The primary etiology of sepsis includes bacterial, fungal, and viral infections (Grondman et al., 2020). Bacteria are the most common cause of sepsis and are mainly divided into Gram-positive and Gram-negative bacteria. Gram-positive bacteria are more prevalent in North America and Europe, while Gram-negative bacteria are more common in Asia (Vincent et al., 2006;Vincent et al., 2009; Sakr et al., 2018). The main bacteria responsible for sepsis are *Staphylococcus aureus* (Gram-positive) and *Escherichia coli* (Gram-negative) (Vincent et al., 2009; Sakr et al., 2018). Viral sepsis has a lower incidence compared to bacterial sepsis, but the high occurrence of viral culture-negative sepsis suggests that there may be many undiagnosed cases of viral sepsis (Lin et al., 2018). The most common viral pathogens are influenza and dengue viruses in tropical regions, both of which can cause seasonal outbreaks, with newborns, children, pregnant women, the elderly, and immunocompromised patients being at risk (Dawood et al., 2012). Fungal sepsis has a higher incidence than viral sepsis but is much lower than bacterial sepsis (Vincent et al., 2006, 2009; Sakr et al., 2018). The main fungal pathogen in sepsis is *Candida albicans*, which is associated with a relatively high mortality rate (Delaloye and Calandra, 2014). Although parasitic infections (<1%) can lead to sepsis, the estimated prevalence within this etiological subclass remains extremely rare (Sakr et al., 2018; Kwizera et al., 2021). The microbiology of sepsis patients can also be classified based on the source of infection, including community-acquired (infections acquired outside of the hospital or any healthcare facility) and hospital-acquired (patients who were infection-free upon admission but developed an infection 48 h or more after admission), with hospital-acquired sepsis being more severe and associated with a higher mortality rate (Westphal et al., 2019).

Sepsis is characterized by two main phases: the hyperimmune phase and the immunosuppressive phase, initiated by the actions of various immune cells triggering a series of immune responses (Nedeva et al., 2019). COVID-19, caused by severe acute respiratory syndrome coronavirus 2 (SARS-CoV-2), involves the invasion of host cells by the virus, leading to failure of host immune recognition. Instead of mounting an effective antiviral immune response upon invasion, the host develops sepsis due to an excessive inflammatory response and suppression of SARS-CoV-2-specific immune reactions (Wang et al., 2022). During the initial hyperinflammatory phase, the upregulation of pro-inflammatory cytokines released by inflammatory cells, along with the activation of the complement and coagulation systems, leads to excessive inflammation, culminating in a cytokine storm and multiple organ dysfunction syndrome (MODS). At this juncture, there is a predominance of neutrophils, activation of lymphocytes, macrophages, and dendritic cells (Chousterman et al., 2017). Concurrently or subsequently, there is an increase in the release of anti-inflammatory cytokines and co-inhibitory molecules, a decrease in HLA-DR expression, immune cell death, and regulatory cell proliferation, resulting in immunosuppression (Hotchkiss et al., 2013). Sepsis-induced immunosuppression stems from both innate and acquired immune dysfunctions, characterized by the release of anti-inflammatory cytokines, immune cell death, T cell exhaustion, and excessive generation of immune regulatory cells, including regulatory T cells (Tregs) and myeloid-derived suppressor cells (MDSCs) (Liu et al., 2022). In sepsis, immunosuppression is closely related to cell anergy, endotoxin tolerance, or immune cell exhaustion. The diminished expression of human leukocyte antigen-DR (HLA-DR) and the upregulation of immune checkpoint molecules, such as programmed cell death protein 1 (PD-1), T cell immunoglobulin and mucin domain-containing protein-3 (TIM-3), as well as B and T lymphocyte attenuator (BTLA), further exacerbate immunosuppression (McBride et al., 2020). Metabolic alterations have emerged as an important driver of immunosuppression inpsis. Studies on the metabolism of T cells in septic patients have revealed changes in the mTOR pathway, leading to an inability to induce glyysis, oxidative phosphorylation, and ATP production. As a result, the lack sufficient energy, impairing not only their functionality but also diminishing their prolifer capacity (Appiah et al., 2021). Furthermore, endotoxin tolerance is considered a mechanism of immunosuppression in sepsis. Endotoxin tolerance refers to a reduced responsiveness of cells to endotoxin (lipopolysaccharide, LPS) stimulation after prior exposure to endotoxin (Liu et al., 2019). The primary features of endotoxin tolerance include the downregulation of inflammatory mediators such as tumor necrosis factor-alpha (TNF-α), interleukin-1β (IL-1β), C-X-C motif chemokine 10 (CXCL10), and the upregulation of anti-inflammatory cytokines like IL-10 and transforming growth factor-beta (TGF-β). Therefore, endotoxin tolerance is often regarded as a regulatory mechanism by the host against excessive inflammation, holding therapeutic significance (López-Collazo and del Fresno, 2013). As septic patients transition into the phase of immunosuppression, disruptions in immune cell functionality ensue, leading to a rapid progression of their condition and a substantial escalation in mortality rates (Boomer et al., 2011).

## 3 The role of immune cells in sepsis

### 3.1 The role of neutrophils in sepsis

**Figure 1:**
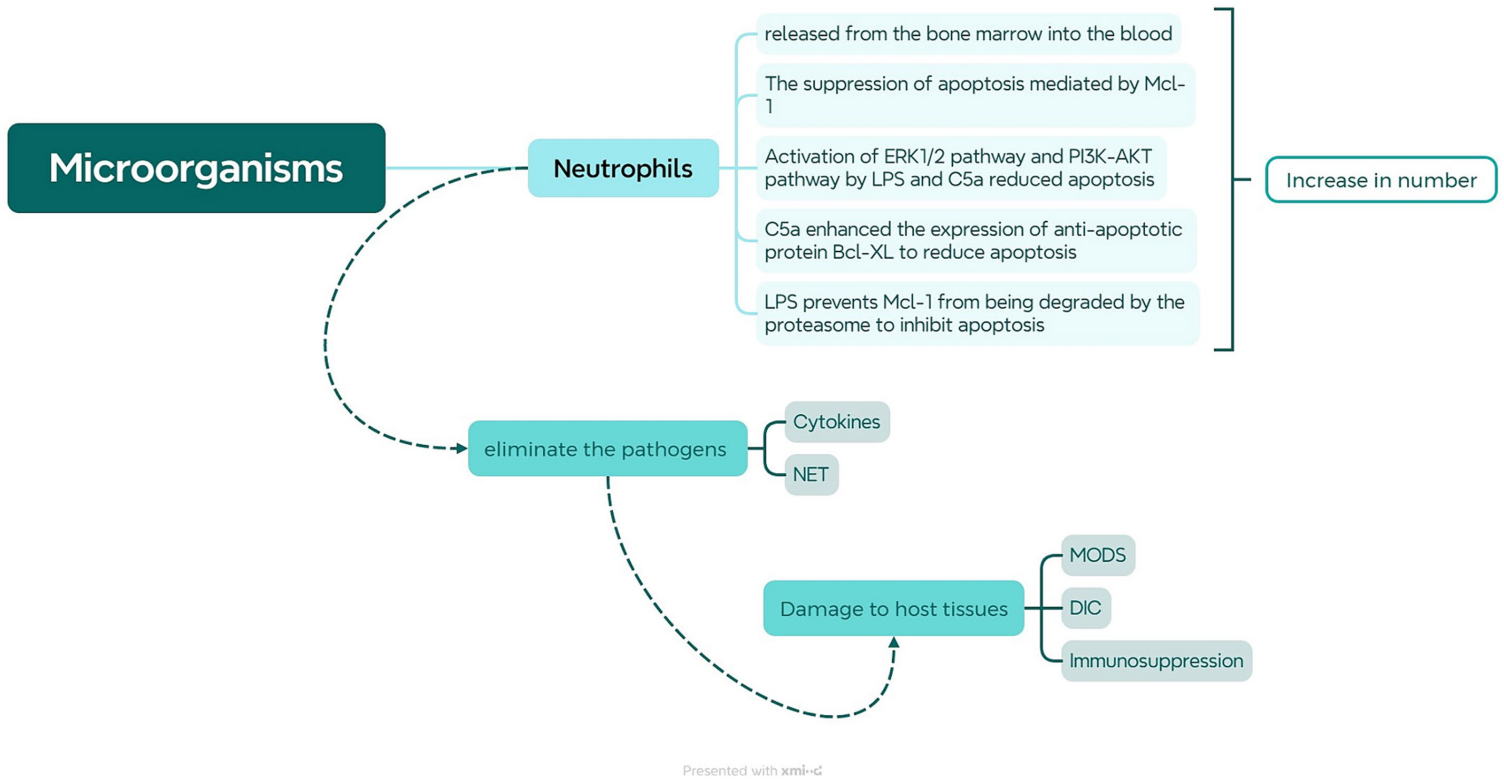
Role of Neutrophils in Sepsis. When microorganisms or their products enter the bloodstream, they stimulate the bone marrow to release neutrophils, causing an increase in neutrophils in peripheral blood. LPS and C5a can activate the ERK1/2 and PI3K-AKT pathways to inhibit neutrophil apoptosis. LPS can also inhibit MNDA cleavage, leading to upregulation of Mcl-1 to suppress neutrophil apoptosis. Furthermore, C5a can upregulate Bcl-xL to suppress Bim expression, thereby inhibiting neutrophil apoptosis. The heightened liberation of neutrophils and reduced apoptosis enable them to more effectively combat pathogens by generating neutrophil extracellular traps (NETs) and releasing cytokines. However, when neutrophils are excessively activated, it may lead to severe consequences such as MODS, DIC, and immune suppression.

In the early stages of infection, the innate immune system is immediately activated, with neutrophils being the primary phagocytes to migrate from the bloodstream to the infection site (Kovach and Standiford, 2012). Neutrophils migrate to the site of infection guided by signals from receptors and chemotactic factors, where they efficiently engulf and eradicate pathogens by releasing reactive oxygen species, antimicrobial proteins, and NETs. Moreover, they can release inflammatory mediators to enhance the immune response. In the context of sepsis, neutrophils are particularly pivotal (Shafqat et al., 2023; Zhang et al., 2023). At this time, a significant influx of neutrophils is observed in the bloodstream, accompanied by inhibited apoptosis and extended half-life, consequently leading to an elevated neutrophil count (as shown in Figure 1). Microorganisms and their products entering the bloodstream can stimulate the large and rapid increase of peripheral blood neutrophils, which can lead to the depletion of the bone marrow neutrophil storage pool and the release of immature cells into the blood (Mare et al., 2015). Immature neutrophils have low recognition and phagocytic ability, cannot effectively remove pathogens, and their poor deformability is more likely to accumulate in capillaries, causing vascular occlusion, tissue hypoxia, and organ damage (Drifte et al., 2013). Cytokines like TNF-α, IL-1β, IL-6, IL-17, and bacterial components can activate G-CSF to promote neutrophil differentiation. Inhibiting the CXCR4/CXCL12 signal axis, G-CSF can promote proliferation and differentiation of CD34+ myeloid progenitors and migration of mature neutrophils from the bone marrow (Delano et al., 2011). Moreover, in individuals with severe sepsis, the significant increase of Mcl-1 can inhibit the apoptosis of neutrophils and promote their life span to increase several times (Milot et al., 2012). Bacterial lipopolysaccharide (LPS) and complement 5a (C5a) can also prolong the life of neutrophils through the following pathways. As LPS and C5a activate ERK1/2, PI3K, and downstream Akt pathways in neutrophils, phosphorylation of Bad inhibits mitochondrial cytochrome C release and reduces apoptosis (Paunel-Görgülü et al., 2012). Furthermore, C5a can reduce neutrophil apoptosis by increasing Bcl-XL expression and reducing Bim expression (Guo et al., 2006). In addition, LPS can inhibit the migration and cleave of MNDA, thereby preventing the degradation of Mcl-1 by proteasome (Fotouhi-Ardakani et al., 2010). The prolonged life of neutrophils can enable them to perform more complex activities in tissues, such as helping to eliminate inflammation or triggering adaptive immune responses, but their persistent presence in tissues may also cause tissue and organ damage.

Over-activated neutrophils produce a large number of bactericidal substances. A variety of complex physiological mechanisms cooperate to fight infections, leading to immune disorders and systemic inflammatory responses, and further inducing coagulation dysfunction and tissue damage (Alves-Filho et al., 2008). Over-activated neutrophils infiltrate and accumulate in important organs, producing a large number of ROS through respiratory burst and releasing bactericidal substances through degranulation, directly or indirectly causing damage to important tissues and organs (Sikora et al., 2021). The generation of oxygen free radicals produced by respiratory burst will cause dysfunction of the mitochondrial transmembrane substance transport system, and the most important is to form calcium overload. And calcium overload destroys the steady state of Ca2+ concentration inside and outside the cell, resulting in a large number of Ca2+ accumulation in the mitochondria, making cells unable to maintain normal function, resulting in mitochondrial, cell, and tissue dysfunction (Mohsin et al., 2021). Additionally, many pro-inflammatory factors and anti-inflammatory factors release induced ROS, leading to an imbalance of oxidation–reduction states in the body, causing oxidative stress reactions and eventually causing organ malfunction. The increased production of ROS may also damage the function of vascular endothelial cells throughout the body, increase vascular permeability, damage mitochondrial function, and eventually cause organ and system dysfunction in individuals with sepsis (Lu et al., 2022). In addition, neutrophils can capture and clear non-phagocytosed pathogens through the production of NETs. NETs are a unique form of cell death in neutrophils, where they release DNA fibers and granules containing antimicrobial proteins to form a web-like structure that ensnares and kills pathogens (Zhang et al., 2023). During the early stage of sepsis, NETs can form a physical barrier that facilitates the capture and clearance of pathogens, preventing their spread and inhibiting the progression of sepsis. However, as the disease progresses, NETs may cause tissue damage, enhanced autoimmunity, and the formation of blood vessel thrombi. This may be attributed to NETs acting as damage-associated molecular patterns (DAMPs), activating the TLR9 receptor to initiate the inflammatory response, promoting the infiltration of inflammatory cells into tissues or organs, and exacerbating tissue damage (Song et al., 2015). Histones are important antimicrobial components of NETs that can also be cytotoxic to endothelial cells, causing endothelial cell damage and affecting microvascular perfusion. They can also promote the generation of thrombin, activate platelets, and inhibit anticoagulants, thereby promoting disseminated intravascular coagulation (DIC) and thrombosis formation (Denning et al., 2019). Neutrophils, through the NET-platelet-thrombin axis, contribute to an increased production of NETs, leading to an increased incidence and mortality rate of sepsis (Allison, 2017). Additionally, the excessive activation of inflammasomes through the Caspase-1-dependent classical pyroptosis pathway induces cell death, causing a massive release of IL-1β and IL-18. Neutrophils infiltrate non-specific organs including liver and kidney, causing the release of inflammatory mediators from damaged cell membranes, thereby amplifying the inflammatory response, accelerating the progression of sepsis, and causing severe tissue injury and organ dysfunction (Liu and Sun, 2019). By the end of sepsis, neutrophils are exhausted, and their migration and function are abnormal, so that neutrophils cannot reach the infection site to control the infection, but accumulate in important organs and cause serious damage (Alves-Filho et al., 2008). ICAM-1 is an adhesion molecule expressed on vascular endothelial cells binds to β2 integrins induced on the surface of neutrophils, is a key molecule in mediating neutrophil rolling adhesion (Dixit et al., 2011). In sepsis, affinity between ICAM-1 and β2 integrins is enhanced, leading cellular rigidification, inducing vascular occlusion and tissue hypoxic injury, which is a significant factor in organ failure. The overexpression of ICAM-in non-specific organ endothelial cells may also be a primary cause of organ tissue damage and functional disruption induced by sepsis (Zhao et al., 2014). Abnormal autophagy and pyroptosis can promote the formation of NET, cause neutrophil membrane damage and release of numerous pro-inflammatory cytokines, and further expand inflammatory response (Zhu et al., 2022). Furthermore, the circulation of immature neutrophils and the emergence of suppressive subsets not only aid in the efficient eradication of infection and hinder the activation and efficacy of other cells like lymphocytes, thereby fostering the development of subsequent immunosuppression (Parthasarathy et al., 2023).

### 3.2 The role of macrophages in sepsis

Macrophages are the most crucial innate immune cells and antigen-presenting cells, possessing high plasticity (Locati et al., 2020). On one hand, they initiate the innate immune response by recognizing risk factors in the microenvironment; on the other hand, they modulate host immune responses through differential polarization, forming a multidimensional phenotypic spectrum in response to microenvironmental changes. Therefore, macrophages play a significant role in regulating host immune balance and inflammatory responses in sepsis (Chen et al., 2021). The primary known phenotypes are inflammatory or classically activated (M1-like) macrophages and healing or alternatively activated (M2-like) macrophages. Each of these polarized macrophage states has distinct functions, and only when they are in balance can the host’s immune homeostasis be maintained (Shapouri-Moghaddam et al., 2018). In the early stages of sepsis, M1-like macrophages can be activated by individual Th1 cytokines (TNF-α and IFN-γ) or pathogen-associated molecular patterns (such as LPS) (Liu et al., 2014; Shapouri-Moghaddam et al., 2018). Recent studies indicate that Caveolin-1 and oxidized low-density lipoprotein also play essential roles in M1-like macrophage polarization (Sivanantham et al., 2023; Wu et al., 2024). M1-like macrophages highly express CD68, CD80, CD86, major histocompatibility complex (MHC)-II, inducible nitric oxide synthase (iNOS), and Toll-like receptor (TLR) 4 (Atri et al., 2018). They increase MHC-II expression by binding to co-stimulatory molecules (CD80 and CD86) and promoting cytotoxic adaptive immunity. The high levels of iNOS in M1-like macrophages contribute to nitric oxide synthesis (Vogel et al., 2014). M1-like macrophages secrete a large number of chemokines (CCL5 and CXCL5) to attract natural killer cells, neutrophils, and activated T cells (Atri et al., 2018). Additionally, M1-like macrophages produce a significant amount of pro-inflammatory cytokines (IL-6, IL-12, IL-23, and IL-1β), reactive oxygen intermediates, and reactive nitrogen intermediates to eliminate host pathogens (Qin et al., 2012). Generally, M1-like macrophages exhibit potent cytotoxic activity, capable of killing pathogens, clearing aberrant endogenous tissues and cells in the immune microenvironment, promoting matrix degradation, and anti-tumor activity (Liu et al., 2021). However, prolonged M1-like macrophage polarization or its enhancement can lead to tissue, organ, and immune cell damage (Chen et al., 2021; Wang et al., 2023). In contrast, during the late stage of sepsis, M2-like macrophages can be activated by Th2 cytokines (IL-4 and IL-13), TGF-β, IL-10, glucocorticoids, and immune complexes (Huang et al., 2018). M2-like macrophages express high levels of C-type lectin (CD206) and scavenger receptor (CD163). They promote the secretion of chemokines (CCL17 and CCL18) to recruit eosinophils, basophils, Th2, and regulatory T cells, exhibiting an anti-inflammatory cytokine spectrum, producing high levels of IL-10, resistin-like alpha (Fizz1), IL-1 receptor antagonist, and TGF-β (Shrivastava and Shukla, 2019). Therefore, M2-like macrophages participate in immune regulation, promote angiogenesis, tissue remodeling, and inflammation suppression (Yunna et al., 2020). Thus, targeted modulation of macrophage polarization and phenotypic alterations as an adaptation to the microenvironment may be an effective therapeutic approach for treating sepsis (Jin et al., 2022).

### 3.3 The role of T cells in sepsis

**Figure 2:**
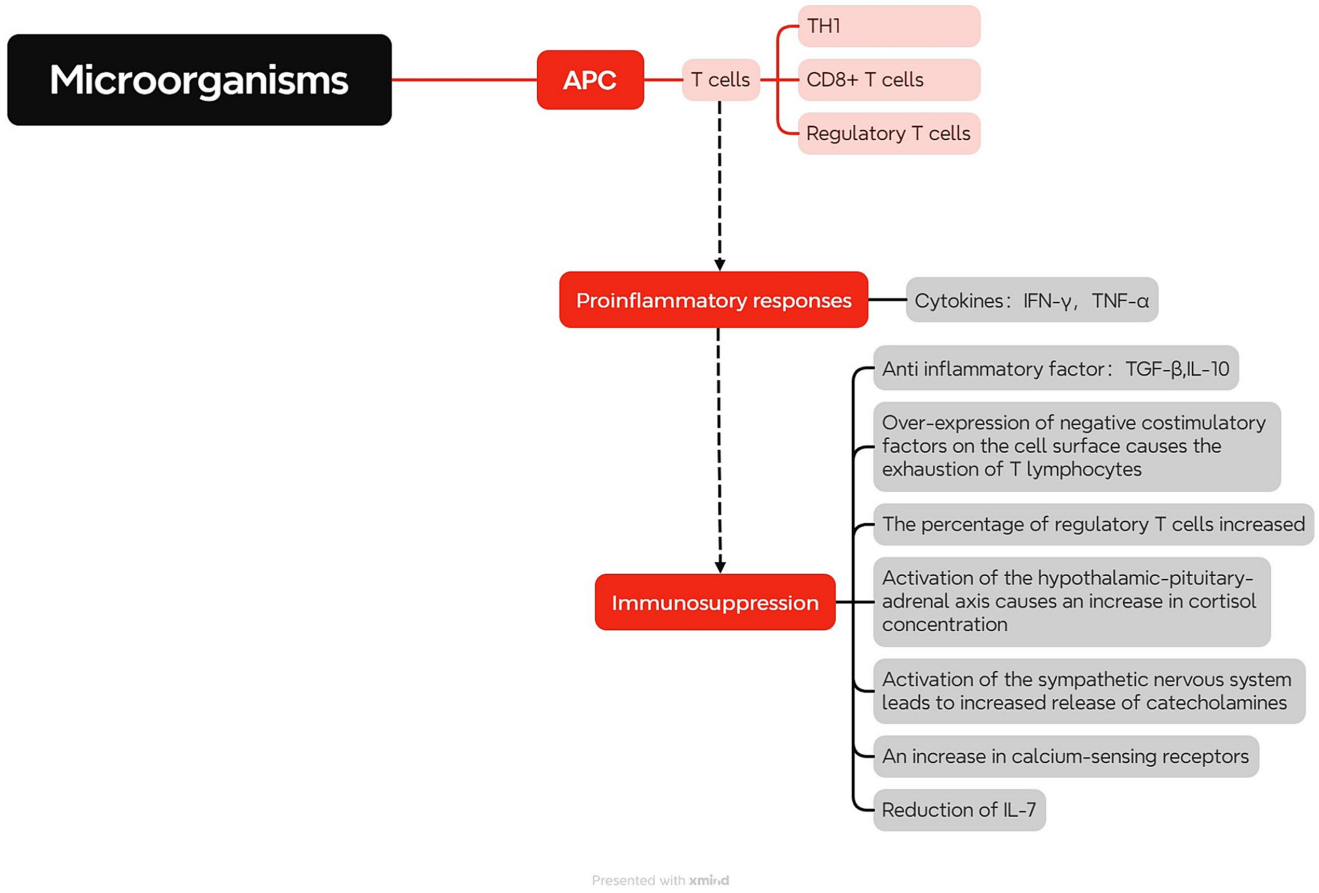
Role of T Cells in Sepsis. Upon microbial invasion, antigen-presenting cells activate T cells, leading to cellular immune reaction. TH1 cells enhance phagocytic activity and bactericidal capacity of macrophages, promote inflammatory reactions, and cellular immune responses by producing cytokines like IFN-γ and TNF-α. CD8+ T cells can recognize and eliminate the infected cells. CD4+ and CD25+ regulatory T cells can inhibit inflammatory response and prevent immune system from damage to their own tissues. However, when T cells are excessively activated, it can activate anti-inflammatory mechanisms, leading to immune suppression and T cell exhaustion. T cell exhaustion is predominantly linked to the excessive expression of inhibitory co-stimulatory receptors on the cell membrane, an upsurge in the proportion of Tregs, activation of the hypothalamic–pituitary–adrenal axis and sympathetic nervous system, heightened expression of calcium-sensitive receptors, and diminished levels of IL-7.

Innate immune cells, beyond their role in phagocytosis and pathogen clearance, can also process pathogens to generate specific antigens and induce adaptive immune responses (Qiu et al., 2019). T lymphocytes, specifically, hold a pivotal position in adaptive immune responses (as shown in Figure 2). Mature T lymphocytes, upon entering the bloodstream, recognize antigens from major histocompatibility complex molecules through surface antibodies. Upon activation and proliferation, they exert their biological functions to eliminate most pathogens (Kasten et al., 2010). TH1 cells enhance the phagocytic activity and bactericidal capacity of macrophages by secreting cytokines like IFN-γ and TNF-α, promoting inflammation and cellular immune responses (Romagnani, 1999). CD8+ T cells identify and eradicate infected cells (Kumar, 2018a). CD4+, CD25+ regulatory T cells modulate immune responses, suppress inflammatory reactions, or prevent damage to self-tissues by the immune system (Siqueira-Batista et al., 2012). However, excessive activation of T lymphocytes can result in the release of substantial quantities of pro-inflammatory mediators, such as IFN-γ, causing excessive inflammation and exacerbating tissue and organ damage. Excessive inflammation triggers anti-inflammatory mechanisms, leading to the production of anti-inflammatory mediators like TGF-β and IL-10 to balance the inflammatory response, resulting in immune suppression characterized by lymphocyte apoptosis and functional inhibition (Yadav and Cartin-Ceba, 2016). When sepsis persists, the patient’s immune function continues to be impaired, leading to profound immune suppression and entering a state of immune paralysis. This can cause sustained organ dysfunction and lead to recurrent infections, even life-threatening conditions (Yang et al., 2014). Research has shown the depletion of T lymphocyte is a major characteristic of immune suppression in sepsis, and there are several mechanisms underlying T lymphocyte depletion in sepsis. The first mechanism involves the overexpression of cell surface negative co-stimulatory factors like PD-1, BTLA, CTLA-4, Tim-3, and LAG-3. These factors inhibit the activation, proliferation, or induction of apoptosis in T lymphocytes, resulting in T lymphocyte depletion. The expression level of cell surface negative co-stimulatory factors is directly associated with the severity of sepsis (Gao et al., 2015; Lange et al., 2017; Lou et al., 2020; Nakamori et al., 2020; Huang et al., 2022). The second mechanism involves an increased percentage of Tregs. Tregs exert immunomodulatory effects in both innate and adaptive immunity and can induce apoptosis in other lymphocytes. Moreover, the expression of PD-1 on Treg cells is directly related to the severity of sepsis (Liu et al., 2017). Additionally, activation of the hypothalamic–pituitary–adrenal axis causes elevated cortisol concentration, which bind to glucocorticoid receptors and exert anti-inflammatory effects. This results in decreased lymphocyte activity and apoptosis (Briegel, 2002). Stimulation of the sympathetic nervous system causes heightened catecholamine, activation of beta-adrenergic receptors, elevated IL-10 secretion, reduced TNF-α secretion, increased release of anti-inflammatory agents, diminished release of pro-inflammatory agents, as well as reduced T lymphocyte activity, proliferation, and apoptosis (Kanczkowski et al., 2016). Other factors, such as increased expression of the CaSR, are also related to T lymphocyte apoptosis in sepsis (Wu et al., 2015). IL-7 play a crucial role in lymphocyte proliferation and maturation. Research has found that of IL-7 levels diminish in individuals with sepsis, resulting in a reduction in T lymphocyte count, with the degree of decline correlating with the severity of sepsis (de Roquetaillade et al., 2018). Therefore, comprehending the influence of T lymphocytes on the immune function of sepsis patients and monitoring changes in T lymphocytes can provide a better understanding of the patient’s immune status and effectively guide clinical interventions (Yuan et al., 2013).

**Figure 3:**
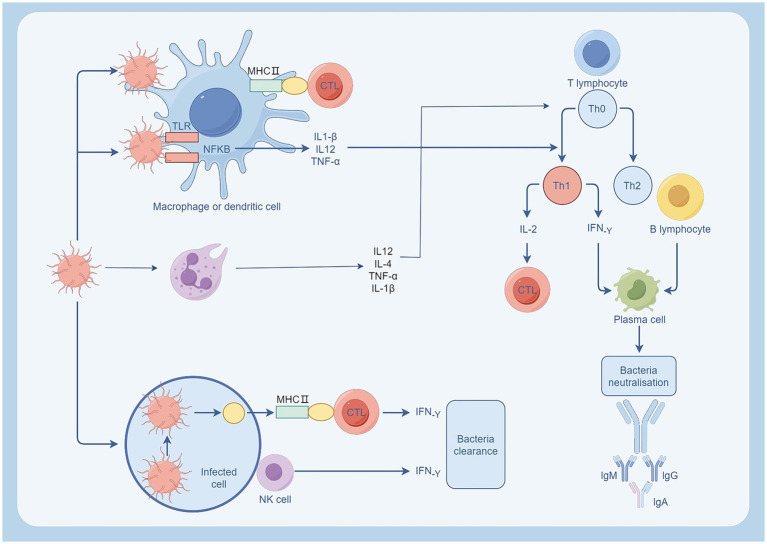
Immune cell response following bacterial infection. Neutrophils and macrophages are activated after bacterial infection. Neutrophils regulate T cells by releasing IL-2, IL-4, and promote inflammation by releasing TNF-α, IL-1β. Macrophages engulf pathogens through phagocytosis and present them to T cells via the MHC II pathway, promoting the differentiation of TH0 to TH1. TH1 cells activate CTL by secreting IL-2 and promote antibody production by plasma cells through the secretion of IF-γ, neutralizing extracellular toxins. Source from: Figdraw.

**Figure 4:**
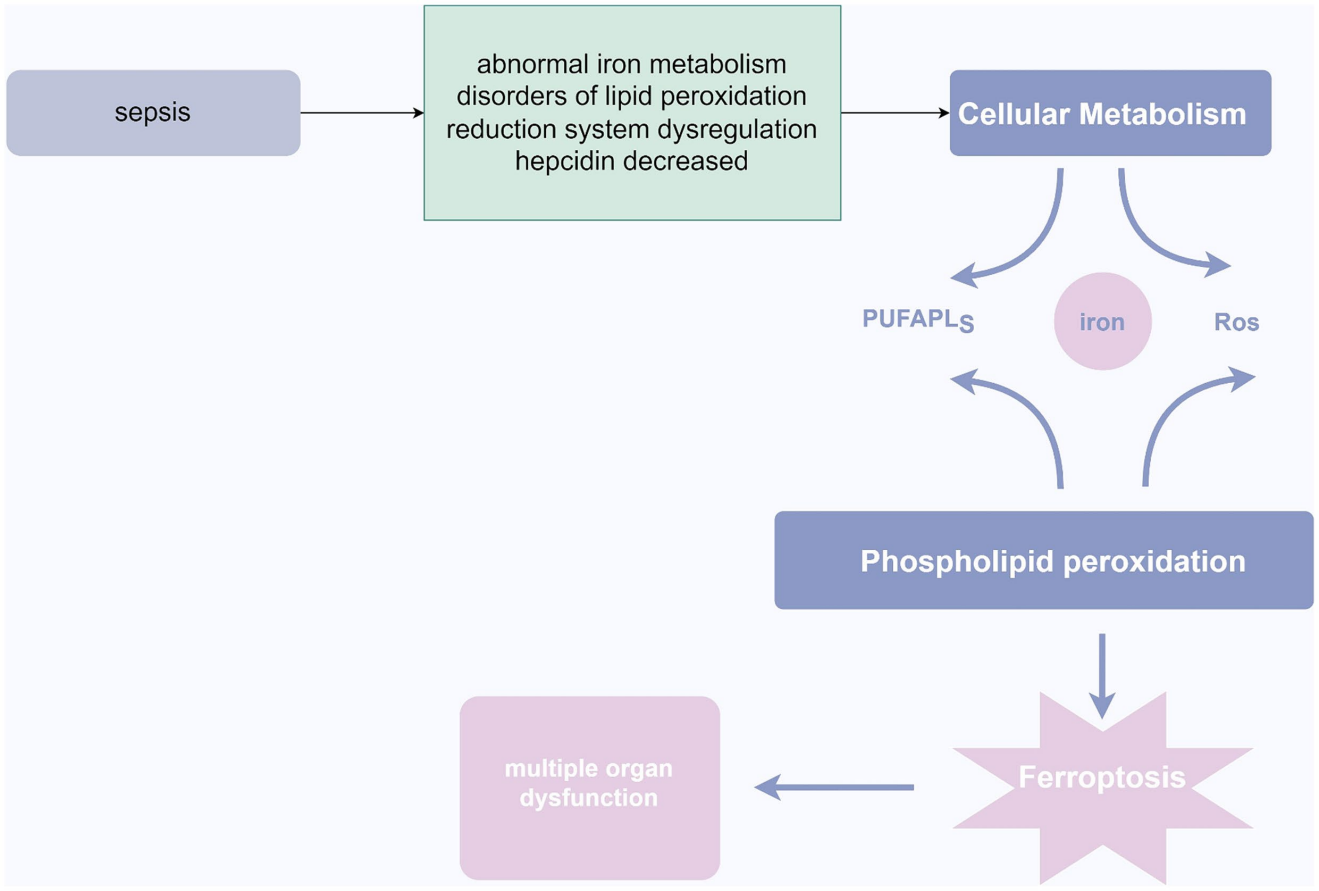
Ferroptosis in sepsis. In the pathogenesis of sepsis, abnormalities in iron metabolism, lipid peroxidation, dysregulation of the redox system, and decreased ferritin levels may lead to cellular metabolic disturbances. This disruption further triggers phospholipid peroxidation, resulting in ferroptosis of cells, ultimately culminating in organ dysfunction. Source from: Figdraw.

In addition, other immune cells also contribute to the body’s immune defense. For example, dendritic cells capture and process pathogens and present their surface antigens to T lymphocytes (Kumar, 2018b). B-cells promote plasma cell production of antibodies to neutralize extracellular toxins (Dong et al., 2023). Furthermore, the interplay among diverse immune cells is a crucial element in the immune response (as shown in Figure 3), including interactions like those between neutrophils and T cells. In addition to antigen presentation, neutrophils also have a regulatory effect on lymphocytes. In sepsis, IFN-γ can induce neutrophils to express PD-L1, and through the PD-L1 signaling pathway, negatively regulate lymphocytes, inhibit their proliferation, activation and release of inflammatory cytokines, and promote lymphocyte apoptosis (Langereis et al., 2017). It can also bind to CD80, competitively obstructing the interaction between CD80 and its ligands, consequently impeding the T cell activation pathway (Sun et al., 2021). In sepsis, neutrophils can also affect the normal cell cycle of T cells by secreting arginase 1, decomposing L-arginine, so that T cells stay in the G0-G1 cycle, resulting in T cell dysfunction (Munder et al., 2006). Moreover, certain neutrophil subpopulations can impede T cell proliferation through Mac-1, suppress the release of IFN-γ, and hinder the activity of T cells (Pillay et al., 2012). During inflammation, T helper (Th) cells differentiate into Th1 cells and Th2 cells, with the equilibrium between the two factions crucial for preserving immune equilibrium (Iwasaka and Noguchi, 2004). IL-12 and IL-4 secreted by neutrophils induce naive CD4 + T cells to differentiate into Th1 and Th2 subtypes, respectively. In sepsis, the amount of IL-4 derived from Th2 is more, and the balance between Th1 and Th2 is imbalanced, presenting an immunosuppressive state (Yoon et al., 2017). T lymphocytes can also activate and enhance the function of neutrophils by secreting cytokines such as IFN-γ, or inhibit the activity of neutrophils by secreting TGF-β, IL-10 (Huang et al., 2022). These interaction and regulation mechanisms are crucial for maintaining immune balance and preventing excessive inflammatory response. In sepsis, the imbalance of the immune system can culminate in an unbridled inflammatory reaction and MODS, so it is important to investigate the interaction and regulation mechanisms between immune cells (Figure 4).

## 4 The role of cytokines in sepsis

Cytokines constitute a vast group of relatively diminutive proteins (<40 kDa) pivotal in cellular signaling, being generated and discharged primarily to facilitate intercellular communication. Cytokines bind to specific receptors on different cell types, inducing activation, proliferation, or migration of target cells. They can be categorized into several groups, including chemokines, interleukins, TNF, interferons, and growth factors (Jarczak and Nierhaus, 2022). In sepsis, the most extensively studied cytokines are TNF-α and IL-1, which can activate target cells and stimulate the production of additional cytokines. Other important cytokines in sepsis include IL-6, IL-8, IL-12, IFN-α, G-CSF, and IL-10 (Chousterman et al., 2017).

TNF is mainly produced by macrophages and lymphocytes. When its concentration reaches a certain threshold, it disrupts the balance of inflammatory reactions, leading to the development of sepsis. There has been significant progress in understanding the mechanism by which TNF causes sepsis, focusing on its ability to induce oxidative damage, abnormal calcium distribution in cells, and activation of caspases (Lendak et al., 2018). TNF-α has the capacity to trigger enzymes like NADPH oxidase and nitric oxide synthase, culminating in the overproduction of reactive oxygen species. The excessive accumulation of these oxygen free radicals can lead to oxidative damage, which can damage cell membranes, proteins and DNA (Blaser et al., 2016). Furthermore, TNF-α has the capacity to facilitate the depletion of intracellular reducing agents like glutathione and glutathione peroxidase, consequently amplifying the magnitude of oxidative harm (Glosli et al., 2002). TNF-α can also promote the release of calcium ions and inhibit their efflux, leading to elevation of intracellular calcium levels. High levels of intracellular calcium can deplete calcium ions within the endoplasmic reticulum, culminating in endoplasmic reticulum stress and abnormalities in protein folding (Duncan et al., 2010). Additionally, high levels of calcium ions can also activate inflammation signaling pathways such as phospholipase A2 and protein kinase C (Dada and Sznajder, 2011). Binding of TNF-α to its receptors can activate signaling pathways like NF-κB and MAPKs, which ultimately activate the caspase-8 and caspase-3, triggering a cascade of apoptosis. Early intervention to block the signaling pathways responsible for inflammatory transmission and inhibition and neutralization of TNF-α have significant implications for the management of sepsis in clinical individuals (Li and Jiang, 2023).

IL-1β, recognized as a cytokine catabolin, belongs to the IL-1 family, comprising 11 genes, and is generated following the activation of the inflammasome, notably NLRP3. IL-1β serves as a pivotal early cytokine in immune modulation and inflammatory reactions, predominantly synthesized by activated monocytes/macrophages, and assumes a significant role in tissue injury (Li and Jiang, 2023). It has great potential in mediating pathological damage, such as activating T cells, stimulating cell production of PGI2, IL-1, IL-6, promoting B cell growth, inducing adhesion molecule expression in endothelial cells, stimulating matrix metalloproteinases and plasminogen activator production in synovial cells, inducing bone resorption, and synthesis of acute phase proteins in the liver (Ge et al., 2019). The collective action of these functions exacerbates systemic inflammatory response, and research has demonstrated that levels of IL-1β are elevated in sepsis non-survivors in comparison to survivors, indicating a relationship between heightened IL-1β levels and sepsis outcomes (Cao et al., 2023). The study of inflammatory mediators has always been an important pathological and physiological aspect in the development of sepsis, as their levels and changes directly reflect the occurrence, development, and prognosis of sepsis (Wang et al., 2018). In recent years, the role of cytokines in the pathogenesis of sepsis-induced organ damage has gained increasing attention from researchers. Targeted therapies directed against inflammatory pathways hold great promise in fundamentally preventing the occurrence and progression of sepsis-induced organ damage.

Some clinical trials have sought to treat sepsis by obstructing certain facets of the inflammatory response, such as tumor necrosis factor and interleukin-1, which are specific inhibitory targets, but the results have often been unsatisfactory. These trials were initiated on the basis of preclinical studies that suggested their efficacy (Takeyoshi et al., 2005). Three pieces of evidence support the notion of cytokine suppression. Firstly, patients with elevated levels of cytokines are more predisposed to mortality. Secondly, experimental animal models indicate that blocking cytokines can ameliorate outcomes. Thirdly, injecting purified recombinant cytokines leads to organ damage and mortality in experimental animals (Remick, 2003). Since the inception of these trials, several other facets of the inflammatory response have been unearthed, with potential new targets including interleukin-18 and HMG-1 (Deng et al., 2022). Nevertheless, prior to commencing new clinical trials, careful consideration must be given as to why previous interventions proved futile. The concept of blocking individual elevated cytokines may be overly simplistic for addressing the intricate issues of sepsis. As patients traverse through various stages of the septic response, there may be appropriate intervals to inhibit multiple cytokines, while at other times, enhancing the immune response may be more fitting (Hotchkiss and Karl, 2001).

## 5 Mechanisms of endothelial cell damage

The main manifestations of sepsis are hypotension, MODS, and DIC. The key pathogenesis of sepsis is endothelial cell injury (Semeraro et al., 2012). When endothelial cells are damaged, fluid leakage from the blood vessels leads to hypotension, ischemia in important organs causes dysfunction, and activation of coagulation factors leads to thrombosis. Impaired endothelial cell junctions and degradation of glycocalyx are key features of endothelial cell injury in sepsis (An, 2009). The connecting structures between endothelial cells are divided into three types: adherent junctions, tight junctions, and gap junctions. These junction complexes play pivotal roles in maintaining tissue integrity, regulating vascular permeability, facilitating leukocyte extravasation, and promoting angiogenesis. When a large number of inflammatory mediators act on endothelial cells, endothelial cell junctions become impaired and vascular integrity is disrupted (Wallez and Huber, 2008). Glycocalyx is composed of membrane-bound domains containing core proteins (such as proteoglycans and glycoproteins bound to oligosaccharides) and plasma proteins (such as albumin and anticoagulants). In the physiological state, its structure and composition remain intact. However, under pathological factors like TNF-α, oxidized lipoprotein, lipopolysaccharide, ischemia/reperfusion, hyperglycemia, or inflammatory stimulation, glycocalyx undergoes degradation and shedding (Foote et al., 2022). Studies have shown that the thickness and integrity of endothelial cell glycocalyx decrease under exposure to lipopolysaccharide and TNF-α (Beurskens et al., 2020). During the progression of sepsis, pro-inflammatory cytokines frequently trigger the activation of mast cells, resulting in the degranulation of mast cells and subsequent liberation of cytokines, histamine, proteases, heparinases, and other glycocalyx-degrading elements. This process damages the endothelial glycocalyx and alters endothelial cell permeability (Becker et al., 2015). On the other hand, glycocalyx shedding exposes integrin and selectin, leading to increased leukocyte adhesion and exudation, endothelial and tissue inflammation, increased vascular permeability, making exudate, albumin and other solutes enter the intercellular space, aggravating tissue edema and reducing blood pressure (Lipowsky, 2018). When the integrity of the blood vessel wall is compromised and stimulated by various microorganisms and their metabolites, endotoxins, inflammatory cytokines, and complement, tissue factor (TF) can be expressed and released by endothelial cells, neutrophils, monocytes, eosinophils, and platelets (Schouten et al., 2008). Upon entering the bloodstream, it activates factor VII and forms a TF/VIIa complex. This complex subsequently activates factor X, catalyzing the conversion of prothrombin into thrombin. Through an expanding positive feedback mechanism, extensive microvascular thrombosis is formed (Konigsberg et al., 2001). During states of inflammation, the body employs three crucial anticoagulant pathways: AT, APC, and TFPI (Okajima, 2001). Under normal physiological conditions, t-PA and u-PA released by endothelial cells serve as primary drivers of fibrinolysis, converting plasminogen to plasmin to break down and eliminate fibrin clots. Simultaneously, endothelial cells can produce the plasminogen activator inhibitor-1 (Grulich-Henn and Müller-Berghaus, 1989). In sepsis, although the levels of t-PA and u-PA increase, but TNF-α and IL-1 may increase the expression of PAI-1, leading to an overall fibrinolysis inhibition (Oszajca et al., 2008). Furthermore, the breakdown of other bodily barriers can also contribute to the progression of sepsis, such as the blood–brain barrier, peritoneal barrier, and others (Grigor'ev et al., 2006; Gao and Hernandes, 2021). Damage to epithelial cells can also lead to sepsis. Epithelial cells line the surfaces of various organs and mucous membranes in the body, such as the respiratory tract, digestive tract, and genitourinary tract. Damage to epithelial cells can allow pathogens to enter the body, triggering infection and subsequent progression to sepsis (Subramanian et al., 2020; Guo et al., 2022; Wang et al., 2024).

## 6 Ferroptosis in sepsis

Ferroptosis is a cell death process that is intracellularly iron-dependent and involves lethal lipid peroxidation reactions, encompassing iron overload, ROS generation, and increased levels of polyunsaturated fatty acids in phospholipids. This leads to loss of cell membrane integrity, disruption of lipid cross-linking affecting normal cell membrane function, and oxidative damage to macromolecules and cell structures, ultimately resulting in cell death (Jiang et al., 2021). The mechanism may involve inhibition of cellular uptake of cysteine, leading to decreased intracellular glutathione (GSH) and inactivation of glutathione peroxidase 4 (GPX4), causing an imbalance in the body’s redox system, accumulation of excessive lipid peroxides, and triggering cell death (Chen et al., 2021). Morphological features include increased cytoplasmic and lipid peroxides, presence of mitochondria smaller than normal in the cytoplasm, with condensed and elevated membrane density, reduced cristae, and rupture of the mitochondrial outer membrane (Wang et al., 2020). Intense stress during sepsis can lead to metabolic disturbances in ions, lipids, and energy (Wasyluk and Zwolak, 2021). Dysregulation of iron homeostasis in the body may result in iron accumulation and abnormal distribution, leading to iron-dependent cell death. Under physiological conditions, excess Fe2+ in cells is oxidized to Fe3+ and stored in ferritin (Tang et al., 2021). However, during sepsis, infection stimulates the upregulation of nuclear receptor coactivator 4 (NCOA4), which specifically recognizes ferritin, initiating ferritin autophagy, releasing a large amount of Fe3+, elevating intracellular free iron concentration, and promoting iron-dependent cell death (Wu et al., 2022). Patients with sepsis can also significantly increase ROS levels through the Fenton reaction, where ROS reacts with polyunsaturated fatty acids (PUFAs) to form toxic lipid peroxides that cause iron-dependent cell death (Du et al., 2023). Under physiological conditions, the body also produces ROS and other oxidants, which are promptly reduced to harmless substances by the body’s reductive system. However, during sepsis, immune system dysregulation leads to imbalance in the reductive system, causing lipid peroxidation disturbances and triggering iron-dependent cell death (Su et al., 2019). Furthermore, during infection, the body produces a large number of inflammatory factors such as IL-6, which inhibits hepcidin production in the liver, leading to increased iron concentration in the blood (Li and Wang, 2023). Iron is a crucial catalyst that promotes oxidative stress reactions, generating large amounts of reactive oxygen species. Excessive production of reactive oxygen species can result in extensive cell death, leading to organ dysfunction and multi-organ failure (Li et al., 2020). Additionally, hyperferritinemia can increase the production of inflammatory mediators and suppress the generation of anti-inflammatory mediators, causing damage to self-tissues through excessive inflammatory reactions (Li et al., 2020; Sun et al., 2020). Studies have shown a significant correlation between elevated serum iron levels, infection markers, lipid peroxidation levels, and increased long-term mortality and incidence of cognitive impairment in sepsis patients (Lan et al., 2018). Therefore, reducing intracellular iron deposition, alleviating inflammatory reactions and lipid peroxidation levels, and blocking the signaling pathways related to iron-dependent cell death may provide new insights into the treatment of sepsis (Li et al., 2020). Increasing evidence suggests that iron ions play a crucial role in anti-inflammation and sepsis, and the effects of drugs targeting iron-related molecules, such as iron ion inhibitors, in sepsis are gradually being confirmed (Xl et al., 2022).

## 7 Progress in the treatment of sepsis

Currently, the clinical treatment of sepsis mainly includes fluid resuscitation, early antimicrobial therapy, and comprehensive therapies such as vasopressors, glucocorticoids, and antimicrobial peptides (Srzić et al., 2022; Wang and Zhang, 2023). Due to the critical role of immune regulation in sepsis, immunotherapy holds great promise and has made significant achievements in the field of oncology. Immunotherapy for sepsis mainly focuses on cytokine modulators, immune checkpoint inhibitors, and anti-apoptosis agents to promote proliferation of immune cells (Liu et al., 2022; Wang and Zhang, 2023). Animal studies have shown promising results for IL-7 and PD-L1, while research on GM-CSF and IFN-γ is ongoing. Cytokine modulators regulate the inflammatory response by promoting pro-inflammatory cytokines or inhibiting anti-inflammatory cytokines (Heming et al., 2016). Lipopolysaccharide combined with IFN-γ can inhibit macrophage autophagy, promote macrophage activation, facilitate bacterial clearance, and improve survival (Patoli et al., 2020). Immune checkpoint inhibitors, such as PD-1/PD-L1, cytotoxic T-lymphocyte-associated antigen-4 (CTLA-4), and indoleamine 2,3-dioxygenase, have been well-established for anti-tumor immunotherapy and hold great potential for sepsis treatment (Zhang et al., 2021; Liu et al., 2022). PD-1/PD-L1 blockade can restore the function of neutrophils, monocytes, T cells, and natural killer (NK) cells in sepsis-induced immunosuppression (Chen and Zhou, 2021). The CTLA-4 pathway is involved in neutrophil-mediated T cell dysfunction in sepsis, and CTLA-4 antibody 33 can improve survival and T cell function in septic mice (Sun et al., 2021). IL-7 induces IFN-γ secretion, promotes T cell proliferation, and inhibits apoptosis (de Roquetaillade et al., 2018). GM-CSF and G-CSF enhance the production of granulocytes and macrophages, suppress cytokine storm, and maintain lung physiology, showing promise as immunomodulators for sepsis with immune paralysis (Venet and Monneret, 2018). As sepsis progresses rapidly, shortcomings in immunomodulatory therapy should be noted. Immunotherapy for tumors may lead to significant immune toxicities, including skin reactions, endocrine disorders, hepatic and renal damage, gastrointestinal toxicity, pneumonia, and rare neurologic and cardiac toxicities (Wesley et al., 2021). Patients with sepsis are prone to experience more severe adverse reactions once they develop these toxicities (Fessas et al., 2020). Furthermore, the use of antibiotics for sepsis treatment may reduce the efficacy of immunomodulators, thus necessitating further research to enhance immunotherapy for sepsis (Gopalakrishnan et al., 2020).

Gene therapy is regarded as one of the most promising new methods for treating diseases (Hattori et al., 2018). Numerous signaling pathways implicated in the inflammatory cascade of sepsis have been elucidated, encompassing NF-κB, JAK/STAT, PI3K/Akt/mTOR, and p38/MAPK (Xu and Chu, 2022). Inhibiting signaling pathways and the expression of downstream genes has emerged as a burgeoning field in sepsis treatment. However, numerous challenges still need to be overcome.

The NF-κB signaling pathway is a classic pathway for studying the pathogenesis of sepsis. NF-κB serves as a pivotal mediator of the inflammatory response and holds significant importance for the development of sepsis (Brown and Jones, 2004). When inactive, NF-κB can associate with inhibitory protein inhibitor of κB (IκB) subunits in the cytoplasm, thereby liberating the IκB kinase complex to impede NF-κB’s binding affinity for nuclear receptors, consequently hindering its migration into the nucleus (Mulero et al., 2019). Nevertheless, upon stimulation, the IκB kinase complex becomes activated, phosphorylating specific sites and lifting the constraint on NF-κB, thus promptly facilitating its translocation into the nucleus and causing the swift release of cytokines like TNF-α, IL-1, IL-6, forming an inflammatory storm (Prescott et al., 2021). Meanwhile, a large amount of inflammatory cytokines can also interact with the NF-κB pathway, creating a feedback loop (Kawai and Akira, 2007). Additionally, NF-κB can regulate apoptosis-related genes, including inhibiting the anti-apoptotic factors Bcl-2 and Bcl-xL and enhanced the pro-apoptotic factors Bax and Caspase (Wu et al., 2021). Excessive activation of the NF-κB pathway and massive apoptosis of macrophages can aggravate the inflammatory response and organ damage, which is a key reason for the high mortality rate in sepsis (Qiu et al., 2019). Therefore, by targeting the NF-κB pathway, regulating the activity of immune cells like macrophages and reducing the levels of NF-κB-driven cytokines like TNF-α, and IL-1, it is plausible to effectively attenuate the inflammatory reaction in sepsis and decrease mortality rates among patients (Li et al., 2023). The following are inhibitors that target the NF-κB pathway to treat sepsis (as shown in Table 1) (Huang et al., 2021; Bahuguna et al., 2022; Fang et al., 2023; Hibbert, 2023; Shan et al., 2023; Zhang et al., 2023; Liu et al., 2024).

**Table 1:** Inhibitors of the NF-κB signaling pathway.

Drugs	Significant	References
Astragaloside Dimethyl Fumarate glucocorticoid MG-132 Paeoniflorin Vitamin C N-acetyldopamine dimer	Inhibition of IKK Inhibition of IKK Induce IKB to inhibit NF-κB pathway Ubiquitin proteasome inhibitor Inhibition of NF-κB expression Inhibits the activity of NF-κB Inhibits the activity of NF-κB	Dada and Sznajder (2011) Li and Jiang (2023) Wang et al. (2018) Takeyoshi et al. (2005) Cao et al. (2023) Remick (2003) Ge et al. (2019)

The JAK/STAT pathway is an intricate signaling cascade that is subject to regulation by a myriad of factors. It plays a crucial role in the pathogenesis of sepsis by participating in the signal transduction of various cytokines and forming a network effect. Due to its unique strength and persistence, the regulatory factors can affect the JAK/STAT pathway from different angles and different target points (Morris et al., 2018). JAKs are members of the Janus soluble tyrosine kinase family associated with receptors without intrinsic kinase activity. The family includes four members: JAK1, JAK2, JAK3, and Tyk2 (Agashe et al., 2022). JAK3 is mainly restricted in hematopoietic cells, while JAK1, JAK2, and Tyk2 are more widely distributed and involved in signal transduction of various cytokines and hormones such as interferon-7 (IFN-7), interleukin (IL), and growth factors (Kisseleva et al., 2002). TNF-α and IL-6 serve not only as markers for assessing the severity and prognosis of sepsis but also as pivotal early inflammatory mediators driving organ dysfunction and mortality (de Bont et al., 1994). In the early stage of infection, cascade reactions are further promoted by the release of cytokines, which accelerate the acute phase inflammatory response of the body, leading to neutrophil adhesion to endothelial cells, activation of the coagulation system, causing the eventual onset of sepsis (Chen et al., 2023). The activation of STAT3 is intricately linked to the release of IL-6 during the acute phase response triggered by endotoxin (Greenhill et al., 2011). The HMGB-1 is a novel late inflammatory mediator implicated the pathogenesis of sepsis. It is an important inflammatory factor causing endotoxin-induced death, has a wide range of extracellular inflammatory effects, and can accelerate the further development of sepsis by inducing and amplifying pro-inflammatory factors (Nogueira-Machado and de Oliveira Volpe, 2012). Research has shown that the JAK/STAT pathway is highly activated during sepsis, and the expression of HMGB-1 mRNA in tissues is significantly enhanced and shows sustained expression (Kim et al., 2009). Therefore, inhibiting the activation of the JAK/STAT pathway can significantly reduce the cascading reaction of inflammatory responses after severe infection. Below are JAK/STAT pathway inhibitors that have been clinically used, but their therapeutic effects on sepsis have not been fully demonstrated (as shown in Table 2) (Li et al., 2020; Verra et al., 2023; Zhang et al., 2023).

**Table 2:** Drugs targeting signaling pathways for sepsis.

Drugs	Targeted	Reference
Tofacitinib Ruxolitinib Baricitinib Dexmedetomidine curcumin Rosavin Astaxanthin	JAK1 and JAK3 JAK1 and JAK2 JAK1 and JAK2 AKT mTOR MAPK MAPK	Lipowsky (2018) Schouten et al. (2008) Konigsberg et al. (2001) Wang et al. (2024) Guo et al. (2022) Li and Wang (2023) Li et al. (2020)

The PI3K/AKT signaling pathway is currently the only known autophagy-inhibiting signaling transduction pathway, which has been confirmed in many studies related to tumors and metabolic diseases. Existing research has also confirmed that this pathway is involved in the regulation of expression of various inflammatory factors in sepsis (Tian et al., 2023). PI3K is a lipid kinase that widely exists in the cytoplasm of various mammalian cells. After receiving signals from tyrosine kinases and G protein-coupled receptors on the cell surface, it recruits the regulatory subunit p85 in close proximity to the plasma membrane. Upon binding with the p85 subunit, the p110 subunit catalyzes the transformation of phosphatidylinositol 4,5-bisphosphate (PIP2), a substrate within the membrane, into phosphatidylinositol 3,4,5-trisphosphate (PIP3). Then, PIP3 binds to the N-terminal PH domain of AKT, triggering the relocation of AKT from the cytoplasm to the cellular membrane. With the assistance of PDK1 and PDK2, AKT is phosphorylated at the threonine phosphorylation site (Thr308) and the serine phosphorylation site (Ser473), culminating in its activation (Ersahin et al., 2015). mTOR, a serine/threonine protein kinase, is evolutionarily conserved and activates AKT. Activated AKT can phosphorylate mTOR, enhancing its activity. mTOR has at least two different catalytic subunits in different complexes, mTORC1 and mTORC2 (Kim and Guan, 2015). Currently, it is believed that activated mTOR exerts autophagy-regulating effects through two pathways. One is the direct phosphorylation of autophagy proteins, as mTOR can phosphorylate various autophagy proteins, blocking the dimerization reaction of ULK1 and hindering the formation of induced autophagosomes, thereby inhibiting autophagy (Al-Bari and Xu, 2020). The other is that mTOR serves as a convergence point for multiple signaling pathways. It has the ability to integrate nutrient and growth factor signals and regulate the cell’s life cycle by promoting transcription and translation (Yang et al., 2022). Interventions targeting different components of the autophagy pathway and modulating the activity of signaling pathways at different stages of the disease may become new therapeutic approaches and breakthroughs for treatment. mTOR is a relatively easily regulated target that has been discovered in tumors and other disciplines. Inhibiting mTOR can effectively activate autophagy, and vice versa (Xu et al., 2020). Currently, PI3K-AKT–mTOR signaling pathway inhibitors are mainly utilized for cancer treatment, and more research is needed for their application in sepsis treatment (as shown in Table 2) (Chen et al., 2022; Rattis et al., 2022).

The MAPK family is a serine/threonine protein kinase that exists in most mammalian cells. It catalyzes the reversible protein phosphorylation and activates the cascade kinase reaction, with a highly conserved molecular structure (Kim and Choi, 2010). The four isoforms of the p38 MAPK family are activated by dual phosphorylation at threonine (T) and tyrosine (Y) sites by MAPK kinases (MKKs). The two sites are separated by one amino acid and form a TGY motif activation loop, further activating downstream cytokines and regulating physiological processes such as inflammation, apoptosis, and oxidative stress (Cuadrado and Nebreda, 2010). Research has shown that the p38 MAPK pathway can regulate the immune cells to release pro-inflammatory cytokines. For example, activated p38 MAPK promotes monocytes to release IL-1 and TNF-α, neutrophils to release IL-8 (Liu et al., 2007). In the cytoplasm, activated p38 MAPK promotes the biosynthesis of TNF-α by upregulating the expression of MAPK-activated protein kinases 2 and 3 proteins (Haddad and Land, 2002). During sepsis, tissue cells are in a stressed state, and the p38 MAPK pathway is easily activated by inflammatory mediators, heat shock, or reactive oxygen species (ROS). ROS can activate downstream cytokines by coupling with Grb2, thereby participating in the activation of the p38 MAPK signaling pathway (Qin et al., 2023). p38 MAPK indirectly activates H3 by phosphorylating MSK1 downstream, and H3 is involved in chromatin formation by binding to DNA in the nucleus. Excessive phosphorylation of H3 can cause chromatin condensation and cell cycle arrest, thereby promoting apoptosis (Kikuchi et al., 2013). p38 MAPK can also lead to the accumulation of p53 beyond a certain threshold, triggering apoptosis by phosphorylating the Ser15 site of p53 (Roy et al., 2018). In summary, the p38 MAPK pathway regulates the progression of sepsis by modulating oxidative stress, the release of inflammatory mediators, and apoptosis. Therefore, inhibiting the activity of the p38 MAPK pathway may become a novel therapeutic approach to treat sepsis (Bauquier et al., 2020). Inhibitors of the p38 MAPK pathway are mainly used to treat cancer, and the drugs for sepsis are still under investigation (as shown in Table 2) (Cai et al., 2021; Gao et al., 2023).

## 8 Conclusion

Here, we summarize the pathophysiological mechanisms and treatment strategies of sepsis, which until now has not been clearly understood because it is indeed complex and individually varies greatly. Numerous studies have shown that sepsis, as a multifactorial disease, is closely related to interactions between immune cells, inflammatory factor storm, endothelial cell injury, and ferroptosis. Molecular biology presents intriguing prospects for sepsis management, offering the potential to impede sepsis progression by targeting the signaling pathways implicated in its inflammatory cascade. However, it is crucial for researchers to enhance their comprehension of the intricate interplay among these fundamental mechanisms and characteristics. Therefore, ongoing and future research is needed to elucidate the relationship between root causes, inducements, and clinical treatment of sepsis, which may develop new therapeutic concepts with more scientific and clinical value.
